# Gender and respiratory factors associated with dyspnea in chronic obstructive pulmonary disease

**DOI:** 10.1186/1465-9921-8-18

**Published:** 2007-03-06

**Authors:** Juan P de Torres, Ciro Casanova, Angela Montejo de Garcini, Armando Aguirre-Jaime, Bartolome R Celli

**Affiliations:** 1 Respiratory Research Unit, Hospital Nuestra Sra de Candelaria, Tenerife, Spain; 2 Pulmonary and Critical Care Division. Caritas-St. Elizabeth's Medical Center, Boston, USA

## Abstract

**Rationale:**

We had shown that COPD women expressed more dyspnea than men for the same degree of airway obstruction.

**Objectives:**

Evaluate gender differences in respiratory factors associated with dyspnea in COPD patients.

**Methods:**

In a FEV_1 _% matched population of 100 men and women with COPD we measured: age, MMRC, FEV_1_, FVC, TLC, IC/TLC, PaO_2_, PaCO_2_, D_LCO_, P_imax_, P_0.1_, Ti/Ttot, BMI, ffmi, 6MWD and VAS scale before and after the test, the Charlson score and the SGRQ. We estimated the association between these parameters and MMRC scores. Multivariate analysis determined the independent strength of those associations.

**Results:**

MMRC correlated with: BMI (men:-0.29, p = 0.04; women:-0.28, p = 0.05), ffmi (men:-0.39, p = 0.01), FEV_1 _% (men:-0.64, p < 0.001; women:-0.29, p = 0.04), FVC % (men:-0.45, p = 0.001; women:-0.33, p = 0.02), IC/TLC (men:-0.52, p < 0.001; women: -0.27, p = 0.05), PaO_2 _(men:-0.59, p < 0.001), PaCO_2 _(men:0.27, p = 0.05), D_LCO _(men:-0.54, p < 0.001), P_0.1_/P_imax _(men:0.46, p = 0.002; women:0.47, p = 0.005), dyspnea measured with the Visual Analog Scale before (men:0.37, p = 0.04; women:0.52, p = 0.004) and after 6MWD (men:0.52, p = 0.002; women:0.48, p = 0.004) and SGRQ total (men:0.50, p < 0.001; women:0.59, p < 0.001). Regression analysis showed that P_0.1_/P_imax _in women (r^2 ^= 0.30) and BMI, DL_CO_, PaO_2 _and P_0.1_/P_imax _in men (r^2 ^= 0.81) were the strongest predictors of MMRC scores.

**Conclusion:**

In mild to severe COPD patients attending a pulmonary clinic, P_0.1_/P_imax _was the unique predictor of MMRC scores only in women. Respiratory factors explain most of the variations of MMRC scores in men but not in women. Factors other than the respiratory ones should be included in the evaluation of dyspnea in women with COPD.

## Background

The influence of gender on the expression of chronic obstructive pulmonary disease (COPD) has received limited attention [1-3].

Dyspnea has been defined as the subjective experience of breathing discomfort consisting in qualitatively distinct sensations that vary in intensity and is derived from interactions among multiples physiological, psychological, social and environmental factors [4]. It is the most important symptom of COPD patients and the main determinant of their Quality of Life (QoL) [5].

In the United States, in the year 2000, more women died from COPD than men [6]. We have recently shown in a population of patients with COPD attending an outpatient clinic, that for the same degree of airway obstruction, women expressed more dyspnea than men at earlier stages of the disease [7].

We therefore hypothesised that systematically studying and comparing different respiratory factors known to contribute to dyspnea in a population of men and women with COPD could help us identify those factors associated with the symptom. Knowledge of these factors, could aid us in the development of tailored strategies aimed at decreasing dyspnea in the female COPD population where this important symptom presents at younger age and earlier stages of the disease. To pursue our goal we called back our patients within the next year of the previous study [7]. At this new appointment we repeated the same original evaluation and also measured other important respiratory factors like the central drive (P_0.1_), the inspiratory and expiratory maximal pressures (P_imax_, P_emax_), the inspiratory capacity to total lung capacity ratio (IC/TLC) and the breathing pattern (respiratory rate and Ti/Ttot).

## Methods

This FEV_1_% case series study, recruited men and post-menopausal women with COPD attending an outpatient clinic at Hospital Universitario Ntra Sra de Candelaria; a tertiary public university hospital in Spain from January 2000 to December 2005. Patients with all degree of airflow severity were included if they had smoked ≥ 20 pack years and had a post-bronchodilator FEV_1_/FVC of <0.7 after 400 micrograms of inhaled albuterol. Patients were excluded if they had a history of asthma, had a history of bronchiectasis, tuberculosis or other confounding diseases. We decided to include only those patients with airway obstruction, therefore patients with GOLD stage 0 were not included. The patients were clinically stable (no exacerbation for at least 2 months) at the time of the evaluation and were part of the BODE international multicenter study [8]. The Ethical Committee of the Hospital approved the study and all patients signed the informed consent.

We evaluated the following parameters in the study sample: age, BMI (weight in kilograms divided by height in meters^2^), ffmi was determined using the bioelectrical impedance Bodystat (Isle of Man, British Isles) and dividing the free fat mass weight in kilograms by height in meters^2^, pulmonary function tests (FEV_1_, FVC, TLC, IC/TLC, FRC, D_LCO_), resting arterial blood gases (PaO_2_, PaCO_2_), dyspnea by the Modified Medical Research Council scale (MMRC) [9] and by the Visual Analog Scale (VAS) [10] immediately before and after the 6-minute walk distance (6MWD test) [11], maximal inspiratory pressures (P_imax_), breathing pattern (respiratory rate, Ti/Ttot, mouth occlusion pressure (P_0.1_), and presence of comorbidities by the Charlson scale [12].

### Pulmonary Function Tests

Postbronchodilator FEV_1 _% of predicted, FVC % of predicted and FEV_1_/FVC, IC, TLC, FRC, D_LCO _values were determined using the European Community for Steel and Coal for Spain as reference values [13] and using a Jaegger 920 MasterLab^® ^Body Box. Inspiratory Capacity was measured immediately before (the best of 3 manoeuvres) and after the 6MWD as previously described [14]. From the lung volume measurements we also determined their IC/TLC ratio.

### P_imax _and breathing pattern measurements

P_imax _was measured in sitting position after 15 minutes of rest from FRC using the technique and predictive values of Black and Hyatt [15]. Breathing pattern was measured also in sitting position after 15 minutes of rest and having carefully explained the manoeuvre to obtain and appropriate measurement. We measured respiratory rate, inspiratory time (Ti), expiratory time (Te) and total breathing time (Ttot).

#### Respiratory drive measurements

The measurement of mouth occlusion pressure (P_0.1_) was performed following the recommendations of Burki et al [16]. To better reflect the central respiratory output of our patients, we calculated the P_0.1_/P_imax _index as we have previously reported [17].

### Data processing

We describe each variable using mean ± SD or median (25^th ^percentile – 75^th ^percentile) depending on their distribution. A multivariate regression analysis with MMRC score as the dependent variable and those parameters that shown to be different between men and female as predictors of its changes was performed. We tested correlations between the MMRC score and the study parameters by non-parametric Spearman's rank or tau-b Kendall's linear correlations coefficients because the ordinal nature of MMRC scales. We then performed a multiple linear regression analysis with MMRC score as the dependent variable and those factors and parameters that shown statistical significant correlation with it (Men: BMI, FEV_1_%, IC/TLC, DLCO, PaO2 and P_0.1_/P_imax_; Women: BMI, FEV_1_%, IC/TLC and P_0.1_/P_imax_). A p value = 0.05 was considered statistical significant. The analysis was performed with the statistical package SPSS^® ^version 12, Chicago, IL, USA.

We describe the matching method as follows: we took our matched patients from an initial sample of 116 males and 59 females with COPD; we were able to matched every female patient with a male with FEV_1 _% of predicted ± 2%; when more than one male matched, we randomly chosen the patient to be included in the final sample, being blind to the rest of the evaluated parameters.

## Results

We were able to match 56 men and 56 women. Four men and six women did not appropriately complete the tests or dropped out of the study. The patients were white of European descent. When enrolled 48% of them were still smoking. Using the GOLD staging system [18], there were the same number of men and women at each stage, distributed as follows: stage I: 25%, stage II: 52%, stage III: 17% and stage IV: 3%.

**Figure 1:**
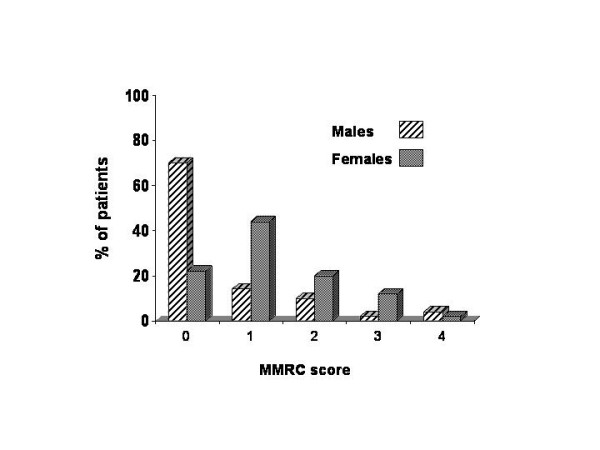
Distribution of MMRC score in COPD men and women. p < 0.05 for the comparison between men and women MRC scores.

Table 1 shows the comparison of clinical and physiological characteristics of the population. Compared with men, women were younger, smoked less, expressed more dyspnea as shown in figure 1, had the same BMI but lower ffmi, a lower diffusion capacity, a higher respiratory center output and walked less in the 6MWD. As expected for an FEV_1_% matched population no differences were found in the other respiratory parameters.

**Table 1:** Clinical and physiologic characteristics of men and women participating in the study.

**Clinical & Physiological Characteristics**	Men* n = 50	Women* n = 50	**p value**
**Age (years old)**	67 ± 8	56 ± 11	<0.001
**Pack years history**	69 ± 27	48 ± 28	<0.001
**MMRC (points)**			
**0 (%)**	35 (70)	11 (22)	<0.001
**1–4 (%)**	15 (30)	35 (78)	<0.001
**BMI (kg/m^**2**^)**	27 ± 4	27 ± 6	ns
**BMI≤21(%)**	5 (10%)	8 (16%)	ns
**ffmi (kg/m^**2**^)**	18 ± 3	15 ± 2	<0.001
**Charlson Score (points)**	2 (2–3)	2(1–4)	ns
**FEV_**1 **_% of predicted**	63 ± 17	63 ± 17	ns
**FVC % of predicted**	91 ± 18	92 ± 21	ns
**FEV_**1**_/FVC % of predicted**	60 ± 12	60 ± 13	ns
**FRC % of predicted**	134 ± 38	134 ± 27	ns
**TLC % of predicted**	113 ± 21	114 ± 21	ns
**IC/TLC (%)**	36 ± 8	36 ± 7	ns
**IC/TLC<0.25 (%)**	6 (12)	4 (7)	ns
**Respiratory rate**	18 ± 4	19 ± 4	ns
**Ti/Ttot**	0,40 ± 0,06	0.41 ± 0.06	ns
**PaO_**2 **_(mmHg)**	76 ± 12	76 ± 9	ns
**PaCO_**2 **_(mmHg)**	41 ± 4	40 ± 4	ns
**DLCO (%)**	87 ± 30	72 ± 20	0.02
**P_**imax **_% of predicted**	67 ± 22	50 ± 20	<0.001
**P**_**0.1**_	0.27 ± 0.11	0.34 ± 0.19	<0.001
**P_**0.1 **_% of predicted**	117 ± 12	155 ± 35	<0.001
**P_**0.1**_/P_**imax**_**	0.04 ± 0.02	0.06 ± 0.05	<0.001
**6MWD (meters)**	522 ± 90	461 ± 85	0.001
**IC/TLC post 6MWD(Lt)**	0.31 ± 0.09	0.31 ± 0.08	ns
**VAS pre 6MWD (cm)**	1 ± 1	3 ± 2	0.05
**VAS post 6MWD (cm)**	3 ± 2	5 ± 2	0.005

mean ± SD or median(25^th^–75^th^ percentiles) depending on scale measurement and sample distribution; ns = statistically non significant.

The multivariate regression analysis with MMRC score as the dependent variable confirmed that only gender (B coefficient: 0.4; 95% CI: 0.1 to 0.8; p = 0.04), age (0.02; 0.007 to 0.032; p = 0.004), p0.1 (1.79; 0.19 to 3.38; p = 0.03) and DLCO (-0.01; -0.02 to -0.007) explained its variations.

Table 2 shows the correlation coefficients between MMRC scores and the studied parameters that reached statistical signification at 0.05 levels. Correlation was found in men with: BMI, FEV_1_%, IC/TLC, DLCO, PaCO_2 _and PaO_2_, P_0.1_/P_imax_, VAS before and after the 6MWD and QoL and in women: BMI, FEV_1_%, IC/TLC,, P_0.1_/P_imax_, VAS before and after the 6MWD and QoL. IC/TLC after the 6MWD correlated with dyspnea after the test in men (-0.36, p = 0.04) and women (-0.40, p = 0.03). No statistically significant correlation was found between MMRC and age or pack year history for women.

**Table 2:** Correlation coefficients of factors that showed significant correlations with the functional dyspnea score as determined with the Modified Medical Research Council scale.

**Clinical and Physiological Parameter**	Correlation coefficient*	
	**Men**	**Women**
**BMI**	-0.29	-0.28
**ffmi**	-0.39	ns
**FEV_**1 **_%**	-0.64	-0.45
**FVC %**	-0.45	-0.33
**IC/TLC**	-0.52	-0.27
**DLCO**	-0.54	ns
**PaO**_**2**_	-0.59	ns
**PaCO**_**2**_	0.27	ns
**P_**0.1**_/P_**imax**_**	0.47	0.46
**VAS pre 6MWD**	0.37	0.52
**VAS post 6MWD**	0.52	0.48

Estimated by Spearman's rank or tau-b Kendall linear correlation coefficients; ns = stastistically non significant

A multiple regression analysis with MMRC as dependent variable and all factors that significantly correlated with it that represent each domain are shown in Table 3. The P_0.1_/P_imax _in women and BMI, DLCO, PaO_2 _and P_0.1_/P_imax _in men had the strongest independent associations with MMRC scores.

**Table 3:** Multiple linear regression with functional MMRC score as dependent variable and those parameters with significant correlation with it. Men included: BMI, FEV_1_%, IC/TLC, DLCO, PaO2 and P_0.1_/P_imax_; Women included: BMI, FEV_1_%, IC/TLC and P_0.1_/P_imax_.

	**Parameter**	**Regression Coefficient**	**95% CI**	**p-Value**
**Men r^**2 **^= 0.81**	**BMI**	-0.70	-0.23 to -0.09	0.001
	**DLCO**	0.32	0.001 to 0.02	0.037
	**PaO_**2**_**	-0.78	-0.08 to -0.05	<0.001
	**P_**0.1**_/P_**imax**_**	0.20	0.16 to 21.6	0.047
**Women r^**2 **^= 0.30**	**P_**0.1**_/P_**imax**_**	0.57	4.9 to 17.1	0.001

## Discussion

To our knowledge this is the first study exclusively designed to study differences in respiratory factors associated to dyspnea in a population sample of men and women with COPD and equivalent degree of airway obstruction. The most important findings are: 1. P_0.1_/P_imax _correlate with MMRC scores in both genders, but was the unique predictor of its scores only in women. 2. Respiratory factors explain most of the variations in MMRC scores in males but not in women with COPD.

Dyspnea is one of the leading symptoms and sometimes the only one affecting patients suffering from COPD. Functional dyspnea has been shown to be a strong predictor of survival [8,19], and an important treatable symptom of the disease [20]. The development of dyspnea in patients with COPD is multifactorial and has been shown to be related to the degree of airway obstruction, pulmonary gas exchange abnormalities, nutritional status, inspiratory muscle strength, lung hyperinflation, respiratory central output, psychological as well as socio-cultural factors [4].

We [7] and others [21] have reported that compared with men, women with COPD report more functional dyspnea for the same degree of airway obstruction. In our initial study we observed that dyspnea appears at earlier stages of the disease in women than in men. In an attempt to find explanations for that observation, we decide to explore the possible association of several of the main respiratory factors thought to be responsible for dyspnea in patients with COPD. We chose patients with mild to severe COPD because this is the group where dyspnea appears to develop earlier in women [7].

Marin et al. have previously determined that central respiratory output is an important factor in the genesis of the dyspnea sensation in patients with severe COPD [22]. Interestingly, in the present study we found that compared with men, women with similar FEV_1_%, FVC%, TLC%, PaO_2_, PaCO_2 _and IC/TLC ratio have a higher central respiratory drive. In the multivariate analysis the P_0.1_/P_imax _was the only independent respiratory factor associated with MMRC scores in women. This is even more important considering that all of our patients were postmenopausal, normoxyemic, normocapnic, well nourished and with a good exercise capacity at the early stages of the disease. Knowing the important stimulant effect that progesterone has on the respiratory center [23] the post menopausal state makes our findings in these patients even more important as lower levels of progesterone tend to decrease the output of the respiratory drive. The exact reason for this association remains unexplored. However, post-menopausal women may manifest unique changes in their physiological responses compared with pre-menopausal women. Indeed, post-menopausal women have less reactive pulmonary vasculature and therefore may develop dyspnea at lower levels of physiological stress compared with pre-menopausal women [24]. New studies addressing these and other mechanisms should provide important useful information.

In our study we also found that the degree of airway obstruction measured by the FEV_1 _and FVC significantly correlated with MMRC score as previously reported by Bestall et al [25]. However, the association between the degree of airway obstruction and degree dyspnea was not strong enough to be retained in the regression analysis models of both men and women. This could be partially explained by the fact that our COPD group is composed mainly of mild to moderate patients, and probably in these stages airway obstruction does not play an important role in the development of dyspnea.

Lung hyperinflation is one of the most important factors related to the development of dyspnea in patients with COPD. It is associated with exertional dyspnea [26] and has also been shown to be an important independent predictor of survival [27]. We found that lung hyperinflation measured by the IC/TLC ratio was associated with functional dyspnea to a similar degree in both genders. We also confirmed in our patients that the degree of air trapped on exertion correlates with the degree dyspnea developed, implying that lung hyperinflation plays an important limiting factor in their activities of daily living.

The importance of arterial levels of O_2 _and CO_2 _in the development of dyspnea in COPD patients is well known [4]. Not surprisingly, their levels as well as the diffusion capacity correlated well with MMRC scores in the male COPD population. Indeed, O'Donnell et al [28] previously reported significant correlation between D_LCO _and dyspnea rating in a population of severely dyspneic COPD patients, even though that the exact physiopathological mechanism is not well described. Interestingly, none of these parameters correlated with MMRC scores in the female patients, implying that probably this is not the most important reason why women with COPD have dyspnea.

Dyspnea has been also associated with the degree of malnutrition specially affecting the respiratory muscles [29]. We confirmed this in our COPD population where we observed an inverse relationship between BMI and MMRC (r = -0.26) similar to that described by Sahebjami et al [29]. This association was not strong enough to remain in the regression model for women, probably implying that malnutrition is not as important in women at these stages of the disease, even considering that they have a lower exercise capacity and Pimax than men with COPD.

We also observed that MMRC and VAS before 6MWD had a strong correlation confirming the face validity of our data and reproducing recent data from Oga et al in a sample of 143 men with COPD [30]. Furthermore, the VAS after 6MWD also correlated with MMRC, implying that the degree of dypnea developed after an exercise test is similar to the one perceived in their activities of daily living as measured by the MMRC, a finding that is similar to that reported by Gallego et al [31].

Very important information is obtained when we analyse the respiratory factors retained in the multivariate regression models for MMRC scores in men and women. The various respiratory factors associated with dyspnea such as nutritional status, carbon monoxide diffusion capacity, level of oxygenation and central respiratory output are responsible for 81% of the variation of MMRC scores in men with COPD. In contrast, we found that the single best predictor of MMRC scores in women, is the central respiratory output but only explaining 30% of its variation. This important finding indicates the need to evaluate novel factors to explain the genesis of dyspnea in women with mild to moderate COPD. Perhaps non respiratory factors such as anxiety, depression or coping mechanisms may play an important role in the perception of dyspnea in women. We support our hypothesis considering that anxiety and depression are highly prevalent in women with COPD and has been shown to be tightly associated with their degree of dyspnea [32] and knowing that men and women with COPD have different coping mechanisms [33]. Our findings also support the findings of Lapperre et al [34] who have suggested that COPD is a heterogeneous disease in terms of its clinical and physiological presentation.

Our study has several limitations. First, the findings here presented should be restricted to a population with similar characteristics of ours, namely men and women with mild to moderate COPD attending an outpatient clinic. It would be interesting to know if these findings are reproducible in populations of severe to very severe COPD patients. We speculate that probably in this group, lung hyperinflation may play a more important role. Second, we did not evaluate anxiety or depression as possible factors associated with dyspnea, but the goal of the study was to only explore the relationship of respiratory factors and dyspnea.

In conclusion, the central respiratory output is associated with functional dyspnea in both men and women with mild to severe COPD who attend an outpatient clinic. The central output was the single best predictor of MMRC scores only in women. Respiratory factors explained most of the variations of MMRC scores in men but not in women. We propose that other factors should be systematically explored in the evaluation of causes of dyspnea in women with COPD. Further studies in women of different age and severity of airflow obstruction are needed to confirm the importance of our findings.

## Competing interests

The author(s) declare that they have no competing interests.

## Authors' contributions

JPT conceived of the study, and participated in its design and coordination and helped to draft the manuscript.

CC participated in its design and coordination and helped to draft the manuscript.

AMG performed the Lung Function Studies and 6MWD.

AAJ performed the statistical analysis.

BC conceived of the study and carried out the critical review of the final manuscript.

All authors read and approved the final manuscript.
